# A Cross-Sectional Study on Hypertension Medication Adherence in a High-Burden Region in Namibia: Exploring Hypertension Interventions and Validation of the Namibia Hill-Bone Compliance Scale

**DOI:** 10.3390/ijerph19074416

**Published:** 2022-04-06

**Authors:** Olivia Nakwafila, Tivani Mashamba-Thompson, Anthony Godi, Benn Sartorius

**Affiliations:** 1 Department of Public Health Medicine, School of Nursing and Public Health, University of KwaZulu-Natal, Durban 4001, South Africa; tivani.mashamba-thompson@up.ac.za (T.M.-T.); benn.sartorius@ndm.ox.ac.uk (B.S.); 2 Department of Public Health, University of Namibia, Oshakati 15001, Namibia; 3 Faculty of Health Sciences, University of Pretoria, Pretoria 0001, South Africa; 4 Department of Biostatistics, University of Ghana, Accra P.O. Box LG 13, Ghana; agodi@ug.edu.gh; 5 Centre for Tropical Medicine and Global Health, Nuffield Department of Medicine, University of Oxford, Oxford OX3 7LG, UK; 6 Department of Health Metrics Sciences, University of Washington, Seattle, WA 98195, USA

**Keywords:** hypertension, adherence, cross-sectional, Hill-Bone compliance scale, interventions, Namibia, LMIC

## Abstract

In Namibia, the prevalence of hypertension among women and men aged 35–64 years is high, ranging from 44% to 57%. In this study, we aimed to determine adherence and predictors to antihypertensive therapy in Khomas region, Namibia. A cross-sectional study was performed to consecutively sample 400 patients from urban and peri-urban settings in Namibia. Results were validated using the Hill-Bone Compliance to High Blood Pressure Therapy Scale. Crude associations between predictors of adherence and compliance were tested using the Pearson chi-square test. A multivariable logistic regression analysis was then performed on adherence variables found to be significant to adjust for confounders, and the results are presented as adjusted odds ratios (aOR) with 95% confidence intervals. A total of 400 patients participated in this study. The participants’ mean age and standard deviation were Mean ± SD = 48.9 ± 12.5. In this study, 351 (87.7%) patients were estimated to have good adherence. Education, employment, and the presence of other chronic diseases were associated with adherence. Following multivariate adjustment, the following factors were significantly associated and are therefore predictors of adherence (95%CI, *p* < 0.005): receiving enough medication at last check-up until next one (OR = 5.44, CI 1.76–16.85), lack of encouragement from family and friends (OR = 0.11 (0.03–0.42)), and attendance of follow-ups on schedule (OR = 8.49, CI = 3.82–18.85). The success of hypertension therapy is dependent on the healthcare systems and healthcare professionals in supplying enough medication, support of friends/family, and maintaining scheduled follow-ups. A combination of interventions using low-cost mobile technology led by healthcare professionals could be endorsed. To fully practice universal access to medication, public and private hospitals in Namibia should collaborate.

## 1. Introduction

Hypertension is the most important leading risk factor for cardiovascular diseases (CVDs), accounting for 17.3 million deaths annually, with 80% of these deaths occurring in low- and middle-income countries (LMICs) [1,2,3]. In Namibia, hypertension among women and men aged 35–64 years is high, ranging from 44% to 57% [4,5]. These prevalence rates are far higher than the global and African regional prevalence of 18% and 27%, respectively [6]. Adherence to therapy is among the many risk factors contributing to hypertension and associated complications. The World Health Organization (2003) defines adherence as the extent to which a person’s behavior—taking medication, following a diet, or executing lifestyle changes—corresponds with agreed recommendations from a healthcare provider [7]. Adherence is a key component to controlling blood pressure (BP) levels and is associated with positive health outcomes, including cost reductions [5,8,9,10]; as well as a reduction in complications, such as stroke (35–40%), heart attack (20–25%), and heart failure (over 50%) [11,12]. Reducing BP could significantly contribute to achieving the World Health Organization (WHO) global target of a 25% relative reduction in the risk of premature mortality from non-communicable diseases (NCDs) by 2025 and the Sustainable Development Goals (SDG) target of a one-third reduction in premature deaths from NCDs by 2030. Conversely, poor adherence has been linked with cardiovascular disease (CVD) complications, concomitant diseases, and higher healthcare costs [13,14]. Additionally, non-adherence increases the patient’s risk of death from 50% to 80% [8]. The evaluation of adherence includes direct and indirect measurement methods, although there is no gold-standard method to measure drug adherence [15]. Equally, several factors are known to influence patients to follow treatment plans optimally, including patient-related factors, healthcare teams, therapy-related factors, and organizational factors [7,16]. In order to improve patient adherence to therapy, targeting barriers and enablers related to each of these factors is essential [7,8].

Indirect measurements of hypertension include but are not restricted to patient interviews, adherence questionnaires, and pill count, whereas direct methods include drug measurement in body fluids and biomarker measurement in body fluids [17]. Adherence questionnaires, such as the Hill-Bone Compliance Scale (HBCHTS) and the Morisky Adherence Questionnaire (MAQ) Scale, are indirect methods [15]. Adherence questionnaires remain valued, although sometimes inaccurate and biased by patients’ behavior (the so-called Hawthorne effect), particularly in a research setting [18]. Direct methods, such as biomarkers, are considered to be more accurate but can be costly [19,20]. Hamididouche et al. stated that an ideal method for monitoring adherence should be reliable, practical, simple, and relatively inexpensive; however, such a method is almost non-existent [18]. Hence, the selection of a method to assess drug adherence should be guided by the objective and the setting of the study [18]. Literature has indicated that non-adherence to chronic medication regimens is common in LMICs [21]. Non-adherence was confirmed in a systematic review and meta-analysis conducted in Ethiopia among 15 countries that used the Morisky Adherence Scale (MMAS) to measure antihypertensive adherence among hypertensive patients [10]. The study found a prevalence of 45% blood pressure non-adherence, of which 83.7% was uncontrolled [10]. Poor BP adherence was also observed in Saudi Arabia and Kwazulu Natal, where less than 50% of blood pressure was controlled due to non-adherence [22,23]. Access to healthcare in Namibia is believed to be good [24]. However, data regarding BP medication adherence in Namibia is minimal, with one study spanning a sample of public health facilities in the Khomas region (capital region) reporting suboptimal adherence in 2017 [5].

Whereas it is substantial to understand the measurement of hypertension adherence, it is equally important to explore different interventions to improve BP medication adherence [5,25,26]. These interventions intentionally target non-adherence contributing factors at different levels: patients, healthcare workers, and the healthcare system [7,23]. Factors influencing adherence to hypertensive medication are specific to each country [27,28]. Education on adherence has been noted as a significant predictor of adherence and its positive impact on clinical outcomes [27,28,29]. Given this background, it is vital to investigate and understand specific barriers to adherence in the current setting. Comorbidities associated with hypertension have also become increasingly high in low- and middle-income countries (LMICs); therefore, there is a need to investigate BP adherence and associated factors [27].

The World Health Organization recommends understanding BP determinants for adherence to reduce the high prevalence of hypertension complications [7]. In choosing a suitable approach, Lam and Fresco (2015) recommend that researchers balance the reliability and practicality of BP instruments for specific settings, considering the purpose of the instrument in order to accurately measure BP adherence [30]. Different instruments, such as the HBCHTS and MMAS, have been developed to measure compliance to antihypertensive therapy [30]. Hence, in this study, we aimed to determine adherence levels to antihypertensive therapy and predictors of adherence to antihypertensive medication in Namibia using ≥80% on the Hill-Bone compliance scale.

## 2. Materials and Methods

### 2.1. Study Design and Participants

We employed a cross-sectional study to assess hypertension adherence among blood pressure patients receiving their BP medication from public and private health facilities in Windhoek district, Khomas region. Khomas region is the capital region of Namibia and, out of the 14 regions, is the most central region in the country [31]. The population in Khomas is 415,780, making it the most populous region in the country [32]. The public health facilities (PHF) are located in rural, urban/CBD, or peri-urban settings, based on their geographical region, degree of urbanization, and economic status. In Namibia, antihypertensive therapy in the public health sector is mainly initiated at a hospital level, with patients accessing follow-up care and medication refills at PHCs and other centers. Private patients are attended by private practitioners and access medication refills from private pharmacies. There are ten (10) public health facilities in Windhoek and six (6) major private medical health centers. The PHCs are located as follows: Groot Aub is located in the rural district, Katutura Health Centre and Robert Mugabe in the central business district (CBD), and the remaining seven health centers in the peri-urban district. We excluded Robert Mugabe clinic because it was utilized as a COVID-19 testing center. The private facilities are distributed across the district as well. Two private health centers were also excluded; one was due to an overload of COVID-19 patients, and the other medical center refused to participate. Hence, the study included BP patients from 9 public health facilities and 4 major private facilities. Details of facilities included in the study are shown in Table 1.

This study included and recruited 400 hypertensive patients diagnosed with hypertension and completed at least one cycle (6 months) of antihypertensive medication refill at the public health center or private centers. Patients were aged 18 years and above and could recognize and tell apart their antihypertensive medicines from any other daily medicines. Blood pressure patients who were not hypertensive or hypertensive but did not complete at least 6 months, patients with mental health diagnoses, and pregnant women were excluded. The primary outcome was the proportion of patients with adherence levels to antihypertensive therapy ≥80% on the Hill-Bone blood pressure scale, and the secondary outcome was the predictors of adherence to hypertensive medication.

The following formula was applied to estimate the sample size:

$$n=\frac{N}{\left(1+Ne^{2}\right)}$$

$n=\frac{236,995}{(1+236,995\;\left(0.05^{2}\right)}$ = 400 participants (total sample size of patients)

where:

n = corrected sample size, N = Population size (based on Namibia Demographic Health Survey (2013)), the percentage of hypertension in Windhoek (WDK) is 57%, and the total population living in WDK is 415,780;

e = margin of error (MOE)  e = 0.05 A two-stage probability sampling method was applied to determine the number of participants from each health facility by ownership (public/private). Considering that up to 80% of the people living in Windhoek sorely depends on public health services [24]; the estimated total sample size was calculated as *n*= 328 (public facilities) and *n* = 72 (private centers). The attendance catchment population of 2019 for each public health facility and their estimated hypertension prevalence during that year was used to estimate the specific sample size for each public facility. As there was no estimated BP (blood pressure) prevalence data from private centers, an equal number of 18 patients was sampled from each private center, namely Roman Catholic, Wanahenda Medical Center, Windhoek West Medical Center, and Pionierspark Medical Centre. A consecutive sampling method was used to recruit participants for this study. Refer to Table 1 for a summary of participants included in the study.

### 2.2. Procedures

#### 2.2.1. Data Collection

Data were collected from 19 January 2021 to 31 June 2021 in Windhoek, Khomas region, using a semi-structured questionnaire. The questionnaire included three (3) sections (A–C). Section A assessed socio—demographic characteristics and anthropometric measurements, section B assessed patient medical history, and section C was the piloted HBCHTS questionnaire (Supplementary File S1). The HBCHTS [33] was used to assess adherence, and it was translated into the most spoken languages in Namibia, namely Oshiwambo, Afrikaans, and English. Trained community health workers (H.C.W.) were used to collect data from the patients, and questionnaires were completed with the assistance of the HCW and the principal investigator. The principal investigator collected the completed questionnaires from the health facilities every day and verified them for clarity and completeness.

#### 2.2.2. Medication Adherence Measures

The HBCHTS reliability (Cronbach’s alpha) coefficient was conducted to measure the consistency of the 14 questions/items. The scale assesses patient behaviors for three important behavioral domains of high blood pressure treatment: reducing sodium intake, appointment keeping, and medication adherence. The higher the Cronbach’s alpha, the better the reliability. The item–rest correlation indicates the correlation between an item and the scale. The items were excluded if the results had a low alpha value and the lowest item–rest correlation. Principal component analysis (PCA) was used to assess construct validity of the entire questionnaire. The Hill-Bone scale was previously validated in Namibia but only for peri-urban areas [5]. Therefore, we felt the need to validate it in the setting of urban and peri-urban areas. The modified Hill-Bone compliance scale shows that many of the patients were compliant (351, 87.7%). All the items except item 5, which asses diet (“how often do you eat fast food “) had the least proportion of respondents who answered none of the time. Among all items, item 12, which assesses how often the patient misses taking their hypertension pills when they feel sick, had the highest proportion of respondents who answered none of the time (92%). Item 14 on how often you miss taking your hypertension pills when you care less scored lowest (0.3%). A summary of descriptive statistics from the HBTS-C questionnaire is presented in Appendix A, Table A1. The modified version of the Namibian HBCHTS is suitable for urban and peri-urban overall. Cronbach’s alpha score for the entire questionnaire was 0.78. The item–rest correlation ranged from 0.232 to 0.642, with all items satisfying the criterion of >0.30. Principal component analysis shows that more than 55% of the variance in the estimation of adherence can be explained with just three components when using the modified HBCHTS version, excluding items 3, 6, and 13. The overall Kaiser–Meyer–Olkin measure of sampling adequacy was 0.8313. Component 1 had entirely positive loadings (correlations) with the 14-item variables, although components 2, 3, and 4 were largely positive. See Appendix A Table A2.

### 2.3. Statistical Analysis

Data were analyzed descriptively and analytically using STATA-17 software. The responses to the 14 original items of the Hill-Bone HBP Compliance to High Blood Pressure Therapy Scale (HB-HBP) were coded as follows: 1 = all the time, 2 = most of the time, 3 = some of the time, and 4 = none of the time, where high values are better. The coding for one variable (item 6) had to be reversed. From the reliability analysis, three (3) variables were dropped. The remaining 11 variables were then summed to give scores on a resulting scale of 11–44, where 11 is the worst score, and 44 is the best. The total scores were then converted to the percentage scale using the following formula:

$$\%\;\mathrm{Adherence}=\left(\frac{\mathrm{Total}\;\mathrm{score}\;\mathrm{obtained}-\mathrm{Lowest}\;\mathrm{possible}\;\mathrm{score}\;\left[11\right]}{\mathrm{Highest}\;\mathrm{possible}\;\mathrm{score}\;\left[44\right]-\mathrm{Lowest}\;\mathrm{possible}\;\mathrm{score}\;\left[11\right]}\right)\times 100$$

The resulting percentage scores were then categorized as good adherence (≥80%) and poor adherence (<80%). This categorized adherence variable was then used in comparisons with respondents’ characteristics and experiences with hypertension using the Pearson chi-square test (or Fisher’s exact test if expected frequency <5 observations). Variables that were significantly associated at 10% level (*p*-value < 0.1) from these bivariate comparisons were inputted into a multivariable logistic regression analysis to determine the unadjusted and adjusted relative odds of having good adherence (≥80%) as opposed to poor adherence.

## 3. Results

### 3.1. Bivariate Analysis of Sociodemographic and Clinical Characteristics

A total of 400 patients participated in this study. Participants who refused to participate were replaced; hence, the response rate was 100%. The participants’ mean age and standard deviation were Mean ± SD = 48.9 ± 12.5. The majority of the participants in this study were female, single, and had at least attended secondary education (50.7%). At least 50% of the participants were employed, and the rest were either retired, unemployed, or were still students. The majority of the participants received a monthly income between N$500–1999. Less than 23% had an income of between N$5000 and N$10,000 or more. Body mass index (BMI) indicates that most patients were overweight and obese, and a few were underweight (Mean ± SD = 28.4 ± 6.7). Fifty-nine (59%) of the patients included in this study had been diagnosed with hypertension for more than five years, and 29% of the total participants had been diagnosed with other chronic diseases and conditions, namely HIV, diabetes, asthma, epilepsy, heart diseases, allergies, high cholesterol level, gastritis, gout, and Hypothyroidism. The patients’ experiences with hypertension and adherence show that more than 80% had an adequate supply of medication to last them until the next visit and had knowledge of the importance of taking medication and the consequences if medication is not taken. Support from family and friends to take medication was more than 70%. Most patients reported that their family and friends did not accompany them during follow-ups (68%). Of the 19% of patients who did not always attend follow-ups, the main reasons were forgetting, transport and financial constraints, distance, laziness, and work commitments. At bivariate analysis, mean systolic blood pressure for those with good adherence (137.0 ± 21.0) mmHg was significantly lower than that for those with poor adherence (149.1 ± 28.0) mmHg; t = 3.61, *p* < 0.001. This was also the case for diastolic blood pressure, where the mean for those with good adherence (86.4 ± 14.4) mmHg was significantly lower compared to those with poor adherence (97.2 ± 19.6) mmHg; t = 4.68, *p* < 0.001. Hypertension adherence across the following items was statistically significant (*p*< 0.001): age, employment, patients receiving adequate medication until the next visit, encouragement of family and friends to take medication, and consistency in attending follow-ups on schedule (see Table 2 and Table 3)

### 3.2. Multivariable Logistic Regression of Adherence on Significance Factors from Bivariate Analysis

A multivariable logistic regression analysis was conducted to control for confounding factors and to identify factors associated with adherence to antihypertensive therapy (adherence > 80%). The following factors were significantly associated with good adherence (95%CI, *p* < 0.005): receiving enough medication at the last check-up until the next one (OR = 5.44, CI 1.76–16.85), encouragement of family and friends (OR = 0.11 (0.03–0.42)), and attendance of follow-ups on schedule (OR = 8.49, CI = 3.82–18.85) (Table 4).

## 4. Discussion

In this study, we aimed to determine the levels of adherence and predictors in antihypertensive therapy in public and private health facilities of a high-burden region in Namibia. In this study, 351 (87.7%) of the patients were estimated to have good BP adherence levels (adherence > 80%). Overall, most of the Hill-Bone items performed well; however, forgetting to take hypertension medication, adherence to dietary advice, missing scheduled appointments, and running out of hypertension medication still raised concerns. The adherence findings of the current study were measured by a modified 11-item validated Hill-Bone compliance (HBCHTS) scale. Sociodemographic characteristics, such as education and employment, were associated with adherence. The presence of other chronic diseases was also associated with adherence. The present study’s findings show that having received enough medication at the last check-up until the next one, family and friends’ encouragement to take medication, and always attending follow-up schedules on time were significantly associated with good adherence and are therefore predictors of BP adherence. High adherence levels are not only good for the control of blood pressure but will also help Namibia achieve the WHO global target of a 25% relative reduction in the risk of premature mortality from NCDs by 2025. Our findings show a need to continue promoting adherence and further strengthening existing policies. This way, Namibia can reach the Sustainable Development Goals (SDG) target of a one-third reduction in premature deaths from NCDs by 2030.

Adherence can vary from region to region depending on the population and the measurement scale [34,35]. It was reported in a study conducted in Palestine that adherence levels in low-income countries range from 30% to 50% and from 50% to 72% in high-income countries [36]. Our study’s findings on adherence are a bit higher than those reported in other low- and middle-income countries [10,21,22,23,37] but very similar to those of a study conducted in the Gaza Strip, Palestine. In the mentioned study (*n* = 521, 80.4%), hypertensive patients were adherent using according to the HBCHTS compliance scale [36]. One of the reasons for the good adherence reported in our study could be attributed to the patients being included in this study. We included patients from private and public health facilities representing low and high economic classes. Existing literature has reported that urban communities are more associated with good adherence compared to rural areas [34]. This could also have been the reason as to why our findings are different from those of a study conducted by Nashilongo et al. (2017) in Namibia in peri-urban settings in the same region (adherence = 48%) [5]. The comparison with Nashilongo’s findings from four health facilities is not entirely surprising because our study consisted of 14 health facilities in total, representing both urban and peri-urban areas of Namibia. A limitation that might have occurred is the aspect of the “Hawthorne effect” [18]. We used community health workers to collect the data, and patients usually tend to respond differently when they believe they are being monitored; as a result, patients may respond untruthfully, which could result in overestimating of results [15,18]. However, in measuring adherence, the health workers were adequately trained, and results from this study were validated. Due to the nature of the study, the variable for the antihypertensive drugs per person was not recorded; however, we captured the number of years the patient had been diagnosed with hypertension. Nonetheless, the current work is the only recent study in Namibia on hypertension adherence noted so far to have been conducted after Nashilongo’s study in 2016.

Studies have shown that some progress has been made to improve adherence by modifying the medication type, dosing, and regimen, with simplified regimens including once-a-day medication promoting best adherence [38,39,40,41,42]. Similarly, in Namibia, the treatment regimen is different for public and private patients, to which our results could be attributed. In this study, we used a self-assessment method to measure adherence. Other studies state that high adherence is attributed to self-assessment methods and improvement of adherence around clinic visits, recall bias, and social acceptability bias, with participants wanting to give socially acceptable answers [27,43]. Based on findings on self-assessment methods, a modified combination of measurements for adherence can be considered, although other authors [44,45] have stated that barriers to adherence might have to be examined by self-assessment and are important in further planning strategies to improve treatment adherence. A couple of studies have confirmed that interventions targeting patient behaviors were more effective than those targeting provider behaviors, such as the Framingham study and another study conducted in Tunisia [46,47]. Nevertheless, healthcare providers retain a key role in modifying patient behaviors to cater to barriers such as forgetfulness and aversion toward medication. Various methods, such as reminders, reviews, information, education, use of phone applications, and motivational interviews [48,49], could be considered as strategies in response to the barriers mentioned in our study, including forgetfulness and avoiding medication.

The World Health Organization has recommended that a strong focus be directed on understanding the variety of factors that may influence antihypertensive medication adherence [23]. In particular, encouragement to regularly take medication is significant. Encouragement can be achieved by using automated cell phone messages set as reminders or electronic methods. Automated cell phone messages can also be used to remind patients when the medication is out of stock; hence, patients can save on unnecessary transport costs. It is also important to collaborate with the private sector, especially pharmacies, to offer affordable medication at private pharmacies, including for patients without health insurance. Health education may also be recommended in the future to encourage patients to not only to take medications when they only feel sick.

When implementing the correct intervention for the right setting, it is equally important to continuously evaluate interventions to constantly improve adherence to treatment regimens [50,51]. We recommend monitoring and evaluating strategies, such as reminder systems for adherence before implementation. In accommodating the aspect of having other chronic diseases, future research should focus on investigating the most effective dosage of hypertension medication, considering patients taking chronic medication for more than one disease. We strongly suggest qualitative research to investigate why individuals are non-adherent and whether universal access to medication in Namibia is fully being put into practice, considering predictors for adherence revealed in this study.

## 5. Conclusions

Overall, adherence to antihypertensive medication was good in patients with a diagnosis of hypertension in Khomas region. Although adherence was good, certain individuals were non-adherent despite universal access to medication in Namibia. The success of hypertension therapy depends on the healthcare system and healthcare professionals in supplying enough medication, support of friends/family, and maintaining scheduled follow-ups. A combination of interventions using low-cost mobile technology led by healthcare professionals could be helpful to further improve adherence. To practice universal access to medication, it may be essential that public and private hospitals in Namibia collaborate.

## Figures and Tables

**Table 1:** Summary of sample size calculation.

Public Facilities	2019 Public Facilities Attendance	% of 2019 Total Public Attendance	Sample Size out of 328 for Public Facilities		
Donkerhoek Clinic	2292	4.1	14	Total sample size for public facilities (82% of 400) = 328	328
Groot Aub Clinic (Windhoek)	2103	3.8	12		
Hakahana Clinic	1851	3.3	11		
Katutura Health Centre	22,008	39.6	130	Total sample size for private facilities (18% of 400) = 72	72
Khomasdal Health Centre	9388	16.9	55		
Maxuilili Clinic	5032	9.0	30		
Okuryangava Clinic	7435	13.4	44	Total sample size for both public and private facilities = 400	400
Otjomuise Clinic	2431	4.4	14		
Robert Mugabe Clinic	-	-	-		
Wanaheda Clinic	3064	5.5	18		
Total	55,604	100.0	328		

**Table 2:** Bivariate analysis of characteristics of respondents and adherence.

	Total	Adherence Score, *n* (%) within Total		
	*n* (%)	<80%	≥80%	*p*-Value
Age (years)				<0.001 ^F^
20–29	22 (5.5)	4 (18.2)	18 (81.8)	
30–39	78 (19.5)	11 (14.1)	67 (85.9)	
40–49	114 (28.5)	21 (18.4)	93 (81.6)	
50–59	96 (24.0)	12 (12.5)	84 (87.5)	
60+	90 (22.5)	1 (1.1)	89 (98.9)	
[Mean ± SD = 48.9 ± 12.5]				
Sex				0.981
Male	130 (32.5)	16 (12.3)	114 (87.7)	
Female	270 (67.5)	33 (12.2)	237 (87.8)	
Marital status				0.897 ^F^
Single	242 (60.5)	29 (12.0)	213 (88.0)	
Married/Cohabiting	138 (34.5)	17 (12.3)	121 (87.7)	
Separated/Divorced	20 (5.0)	3 (15.0)	17 (85.0)	
Education				0.018 ^F^
None	21 (5.3)	2 (9.5)	19 (90.5)	
Primary	117 (29.2)	8 (6.8)	109 (93.2)	
Secondary	203 (50.7)	25 (12.3)	178 (87.7)	
Tertiary	59 (14.8)	14 (23.7)	45 (76.3)	
Employment				<0.001 ^F^
None/Student	104 (26.0)	13 (12.5)	91 (87.5)	
Employed	168 (42.0)	32 (19.1)	136 (81.0)	
Self-employed	35 (8.8)	3 (8.6)	32 (91.4)	
Retired	93 (23.2)	1 (1.1)	92 (98.9)	
Income				0.013
<500	81 (20.3)	7 (8.6)	74 (91.4)	
500–1999	148 (37.0)	11 (7.4)	137 (92.6)	
2000–4999	89 (22.2)	14 (15.7)	75 (84.3)	
5000–9999	44 (11.0)	7 (15.9)	37 (84.1)	
10,000+	38 (9.5)	10 (26.3)	28 (73.7)	
BMI (kg/m^2^)				0.107 ^F^
Underweight	10 (2.5)	1 (10.0)	9 (90.0)	
Normal	129 (32.3)	19 (14.7)	110 (85.3)	
Overweight	122 (30.5)	8 (6.6)	114 (93.4)	
Obese	139 (34.7)	21 (15.1)	118 (84.9)	
[Mean ± SD = 28.4 ± 6.7]				
Total	400 (100.0)	49 (12.3)	351 (87.7)	

^F^: Fisher’s exact test *p*-value; all others from Pearson Chi-square.

**Table 3:** Bivariate analysis of experiences with hypertension and adherence.

	Total	Adherence Score, *n* (%) within Total		
	*n* (%)	<80%	≥80%	*p*-Value
Years with hypertension				0.367
<5	164 (41.0)	23 (14.0)	141 (86.0)	
5+	236 (59.0)	26 (11.0)	210 (89.0)	
Have other chronic illness				0.014
No	283 (70.7)	42 (14.8)	241 (85.2)	
Yes	117 (29.3)	7 (6.0)	110 (94.0)	
Received enough medication at last check-up until next one				<0.001 ^F^
No	30 (7.5)	10 (33.3)	20 (66.7)	
Yes	370 (92.5)	39 (10.5)	331 (89.5)	
Medics tell when, how, and need for medication				0.369
No	76 (19.0)	7 (9.2)	69 (90.8)	
Yes	324 (81.0)	42 (13.0)	282 (87.0)	
Know the consequences of not taking medication				0.442
No	55 (13.8)	5 (9.1)	50 (90.9)	
Yes	345 (86.2)	44 (12.8)	301 (87.2)	
Family and friends encourage taking of medication				<0.001
No	106 (26.5)	3 (2.8)	103 (97.2)	
Yes	294 (73.5)	46 (15.7)	248 (84.3)	
Family and friends provide company during follow-ups				0.424
No	274 (68.5)	36 (13.1)	238 (86.9)	
Yes	126 (31.5)	13 (10.3)	113 (89.7)	
Always attend follow-ups on schedule				<0.001
No	74 (18.5)	25 (33.8)	49 (66.2)	
Yes	326 (81.5)	24 (7.4)	302 (92.6)	
Total	400 (100.0)	49 (12.3)	351 (87.7)	

^F^: Fisher’s exact test *p*-value; all others from Pearson Chi-square.

**Table 4:** Multivariable logistic regression of adherence on significant factors from bivariate analysis. Variables with *p*-values < 0.1 from the chi-square tests compared to adherence. Dependent variable: adherence scores (relative odds of good adherence ≥80% vs. <80%).

	Unadjusted		Adjusted	
	OR (95% CI)	*p*-Value	OR (95% CI)	*p*-Value
Age (years)	1.05 (1.02, 1.08)	<0.001	1.01 (0.97, 1.06)	0.524
Education				
None	Ref		Ref	
Primary	1.43 (0.28, 7.28)	0.663	3.59 (0.53, 24.21)	0.189
Secondary	0.75 (0.16, 3.41)	0.709	1.53 (0.26, 9.08)	0.642
Tertiary	0.34 (0.07, 1.64)	0.178	1.13 (0.16, 8.07)	0.906
Employment				
None/Student	Ref		Ref	
Employed	0.61 (0.30, 1.22)	0.161	3.35 (0.61, 18.24)	0.162
Self-employed	1.52 (0.41, 5.70)	0.531	4.33 (0.64, 29.24)	0.133
Retired	13.14 (1.68, 102.55)	0.014	27.85 (1.98, 391.46)	0.014
Income				
<500	Ref		Ref	
500–1999	1.18 (0.44, 3.17)	0.745	0.47 (0.12, 1.83)	0.279
2000–4999	0.51 (0.19, 1.33)	0.166	0.18 (0.02, 1.26)	0.083
5000–9999	0.50 (0.16, 1.53)	0.225	0.20 (0.02, 1.66)	0.134
10,000+	0.26 (0.09, 0.76)	0.014	0.10 (0.01, 0.84)	0.034
Have other chronic illness	2.74 (1.19, 6.29)	0.018	1.87 (0.72, 4.85)	0.201
Received enough medication at last check-up until next one	4.24 (1.85, 9.72)	0.001	5.44 (1.76, 16.85)	0.003
Family and friends encourage taking of medication	0.16 (0.05, 0.52)	0.002	0.11 (0.03, 0.42)	0.001
Always attend follow-ups on schedule	6.42 (3.40, 12.13)	<0.001	8.49 (3.82, 18.85)	<0.001

## Data Availability

The data analyzed during the current study are available from the corresponding author on reasonable request.

## Supplementary Materials

The following are available online at https://www.mdpi.com/article/10.3390/ijerph19074416/s1, File S1: Blood pressure medication adherence questionnaire consisting of Section A: Socio-Demographic characteristics and Anthropometric measurements, Section B: Personal Medical History and Section C: Hill-bone Compliance to High Blood pressure therapy Scale.

## Appendix A

**Table A1:** Hill-Bone medication adherence summary statistics and reliability.

				Hill-Bone (14-Item)		Modified (11-Item)	
#	Item (Scoring: 1 = All the Time, 4 = None of the Time	Mean	SD	Item-Rest Correlation	Cronbach Alpha	Item-Rest Correlation	Cronbach Alpha
	How often they:						
1	forget to take hypertension medicine	3.6	0.6	0.413	0.653	0.467	0.767
2	decide not to take hypertension medicine	3.8	0.5	0.428	0.654	0.452	0.768
3	eat salty food	3.0	0.8	0.147	0.696		
4	shake salt on food before eating	3.7	0.6	0.264	0.673	0.246	0.794
5	eat fast food	3.2	0.6	0.234	0.677	0.232	0.795
6	do not make the next appointment before leaving the doctor’s office	2.9	1.3	0.083	0.760		
7	miss scheduled appointments	3.7	0.6	0.448	0.650	0.503	0.762
8	forget to get prescriptions filled	3.7	0.6	0.450	0.648	0.491	0.764
9	run out of high blood pressure pills	3.7	0.5	0.320	0.667	0.288	0.785
10	skip hypertension medicine before going to the doctor	3.8	0.4	0.619	0.643	0.642	0.754
11	miss taking hypertension pills when feeling better	3.8	0.5	0.513	0.647	0.598	0.753
12	miss taking hypertension pills when feeling sick	3.9	0.5	0.442	0.656	0.509	0.763
13	take someone else’s hypertension pills	4.0	0.2	0.109	0.687		
14	miss taking hypertension pills when caring less	3.9	0.4	0.582	0.649	0.626	0.757
	Overall				0.685		0.786

**Table A2:** Principal Component Analysis for construct validity.

	Hill-Bone (14-Item)			Modified (11-Item)		
Component	Eigenvalue	Proportion of Variance	Cumulative Variance	Eigenvalue	Proportion of Variance	Cumulative Variance
1	3.9996	0.2857	0.2857	3.9462	0.3587	0.3587
2	1.4112	0.1008	0.3865	1.1150	0.1014	0.4601
3	1.2103	0.0865	0.4729	1.0428	0.0948	0.5549
4	1.1033	0.0788	0.5517			
