# Supplementary material for: A Cross-Sectional Study on Hypertension Medication Adherence in a High-Burden Region in Namibia: Exploring Hypertension Interventions and Validation of the Namibia Hill-Bone Compliance Scale
Source: Int J Environ Res Public Health. 2022 Apr 6;19(7):4416. doi: 10.3390/ijerph19074416 (PMC8998252; doi:10.3390/ijerph19074416)
Supplement: Supplementary file 1 [file ijerph-19-04416-s001.zip › ijerph-1624479-supplementary.pdf]

**Title: A cross-sectional study on hypertension medication adherence in a high burden region in Namibia: Exploring hypertension interventions and validation of Namibia Hill-Bone scale**

Supplementary File S1: Blood pressure medication adherence Questionnaire.

**Section A: Socio- Demographic characteristics and Anthropometric measurement.**

| **Socio Demographic Characteristics** | |
|---|---|
| **1. Age** | |
| **2. Sex** | Male ☐<br><br>Female ☐ |
| **3. Marital Status** | Single ☐<br>Married ☐<br>Separated ☐<br>Divorced ☐<br>Cohabitating ☐ |
| **4. Highest level of education** | Primary ☐<br><br>Secondary ☐<br><br>Tertiary ☐<br><br>None ☐ |
| **5. Employment status** | Student ☐<br><br>Employed ☐<br><br>Self-employed ☐<br><br>Unemployed ☐ |
| **6. Income level** | Between N$500-N$2000 ☐<br><br>More than N$2000-N$5000 ☐<br><br>More than N$5,000 - N$10000 ☐<br><br>More than N$10000-N$20000 ☐<br>More than N$ 20000 ☐ |
| **Anthropometric measurements** | |
| 1. Height (m)<br>2. Weight (kg) | |
| 3. Blood pressure(mmHg) | |

**SECTION B: Personal Medical History.**

1. When were you diagnosed with High blood pressure? (Year) ____________________

2. Do you have any other chronic illness (es) _____________________________________?

3. If yes, please mention it (them) _____________________________________________

4. At your last visit at the clinic for your high blood pressure follow-up, did you receive enough? medication to last you until your next visit?

_____________________________________________________________________

5. Did your nurse or doctor tell you when, how and why you need to take your high blood pressure medication every time you are supposed to?

_____________________________________________________________________

6. Do you know what might happen if you do not take your medication every time you are supposed to? ____________________________________________________________

7. Do your friends and family encourage you to take your medication?

______________________________________________________________________

8. Do your friends and family accompany you to the clinic for follow-ups?

______________________________________________________________________

9. Do you always go to your follow-ups on the days you are supposed to?

_____________________________________________________________________

10. if not, please say why? -

_________________________

**This section of the questionnaire is adopted from** [1]

**SECTION C:** Hill-Bone Compliance to High Blood Pressure Therapy Scale.
(*Please answer the following questions in the table below, by marking with an (X) appropriately*.)

1. How often do you forget to take your hypertension medicine?
**None of the time** ☐ **Some of the time** ☐ **Most of the time** ☐ **All the time** ☐

2. How often do you decide not to take your hypertension medicine?
**None of the time** ☐ **Some of the time** ☐ **Most of the time** ☐ **All the time** ☐

3. How often do you eat salty food?
**None of the time** ☐ **Some of the time** ☐ **Most of the time** ☐ **All the time** ☐

4. How often do you shake salt (uncooked salt) on your food before you eat it?
**None of the time** ☐ **Some of the time** ☐ **Most of the time** ☐ **All the time** ☐

5. How often do you eat fast food (porsie chips, fat cooks, kapana etc.)
**None of the time** ☐ **Some of the time** ☐ **Most of the time** ☐ **All the time** ☐

6. How often do you make the next appointment before you leave the doctor`s office?
**None of the time** ☐ **Some of the time** ☐ **Most of the time** ☐ **All the time** ☐

7. How often do you miss scheduled appointments?
**None of the time** ☐ **Some of the time** ☐ **Most of the time** ☐ **All the time** ☐

8. How often do you forget to get prescriptions filled?
**None of the time** ☐ **Some of the time** ☐ **Most of the time** ☐ **All the time** ☐

9. How often do you run out of high blood pressure pills?
**None of the time** ☐ **Some of the time** ☐ **Most of the time** ☐ **All the time** ☐

10. How often do you skip your hypertension medicine before you go to the doctor?
**None of the time** ☐ **Some of the time** ☐ **Most of the time** ☐ **All the time** ☐

11. How often do you miss taking your hypertension pills when you feel better?
**None of the time** ☐ **Some of the time** ☐ **Most of the time** ☐ **All the time** ☐

12. How often do you miss taking your hypertension pills when you feel sick?
**None of the time** ☐ **Some of the time** ☐ **Most of the time** ☐ **All the time** ☐

13. How often do you take someone else's hypertension pills?
**None of the time** ☐ **Some of the time** ☐ **Most of the time** ☐ **All the time** ☐

14. How often do you miss taking your hypertension pills when you care less?
**None of the time** ☐ **Some of the time** ☐ **Most of the time** ☐ **All the time** ☐

1. Nashilongo MM, Singu B, Kalemeera F, Mubita M, Naikaku E, Baker A, et al. Assessing Adherence to Antihypertensive Therapy in Primary Health Care in Namibia: Findings and Implications. Cardiovasc Drugs Ther. 2017 Dec;31(5-6):565-78. PMID: 29032396. doi: 10.1007/s10557-017-6756-8.
